# Supplementary material for: The Therapeutic Effect and the Potential Mechanism of Flavonoids and Phenolics of Moringa oleifera Lam. Leaves against Hyperuricemia Mice
Source: Molecules. 2022 Nov 25;27(23):8237. doi: 10.3390/molecules27238237 (PMC9738809; doi:10.3390/molecules27238237)
Supplement: Supplementary file 1 [file molecules-27-08237-s001.zip › molecules-2042254-supplementary.pdf]

The Therapeutic Effects and the Potential Mechanism of Flavonoids and Phenolics of *Moringa oleifera* Leaves Against Hyperuricemia mice

Xiaowei Luo, Lanzhen Zhang[a,*]

[a] *School of Chinese Pharmacy, Beijing University of Chinese Medicine, Beijing 102488, China.*

Table S1 Identification of compounds in MOL-FP by UPLC-Q-Exactive-MS/MS.

Number	RT (min)	Compound	Calculated $[M-H]^+$ (*m/z*)	Observed $[M-H]^+$ (*m/z*)	Molecular formula	Observed MS2 (m/z)
A	5.4	Chlorogenic acid	353.0878	353.0883	C16H18O9	191.0562, 179.0351, 161.0245, 135.0452
B	6.25	Marumoside B	458.1667	458.167	C20H29NO11	504.1729, 307.1038, 150.0549
C	9.82	Isovitexin	431.0987	431.0973	C21H20O10	269.0456, 311.0565, 341.0668, 353.0667, 413.0884
D	10.08	Isoquercetin	463.0881	463.0883	C21H20O12	463.0883, 301.0341, 283.0522
E	10.73	3-p-Coumaroylquinic acid	337.0928	337.0931	C16H18O8	337.0931, 163.0389
F	11.11	Astragalin	447.0937	447.0926	C21H20O11	225.9275, 255.0318, 285.0386
G	12.13	5-p-Coumaroylquinic acid	337.0928	337.093	C16H18O8	337.0930, 163.0389
H	12.62	Niazirin	278.1033	278.1031	C14H17NO5	324.1089, 146.8826
I	13.39	Vicenin-2	593.1512	593.1516	C27H30O15	593.1516, 575.1323, 353.0670
J	14.22	Catechin	335.0772	335.0776	C15H14O6	335.0776, 146.9374, 137.0232, 133.0282

[*]Corresponding author: E-mail address: zhanglanzhen01@126.com (Lanzhen Zhang).
Tel.: +86 13699205903

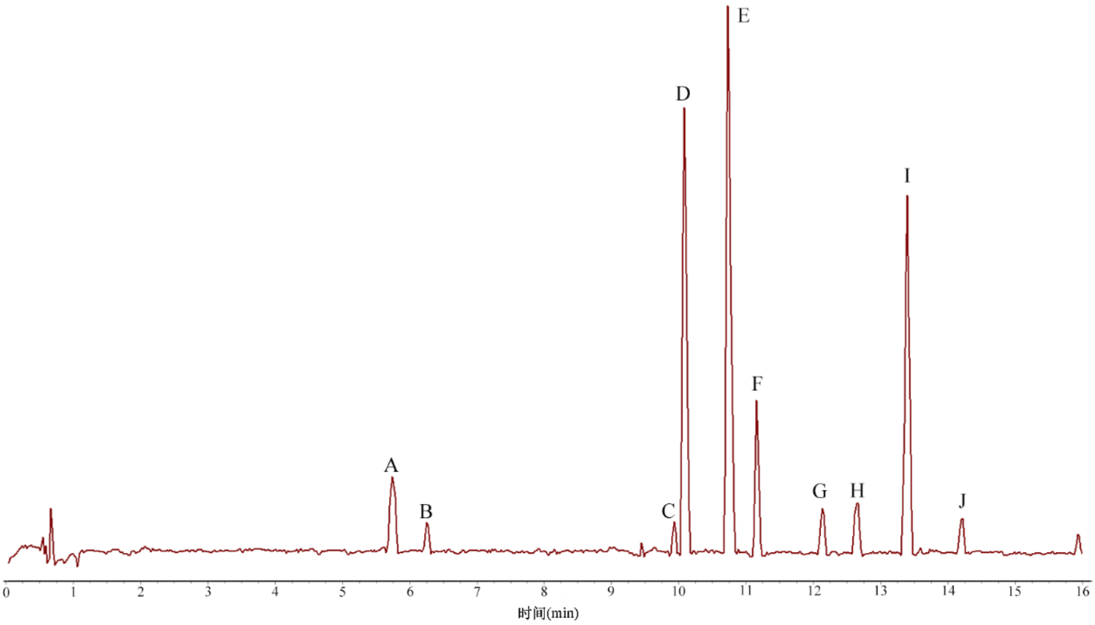

Figure S1. Total ion chromatograms of MOL-FP

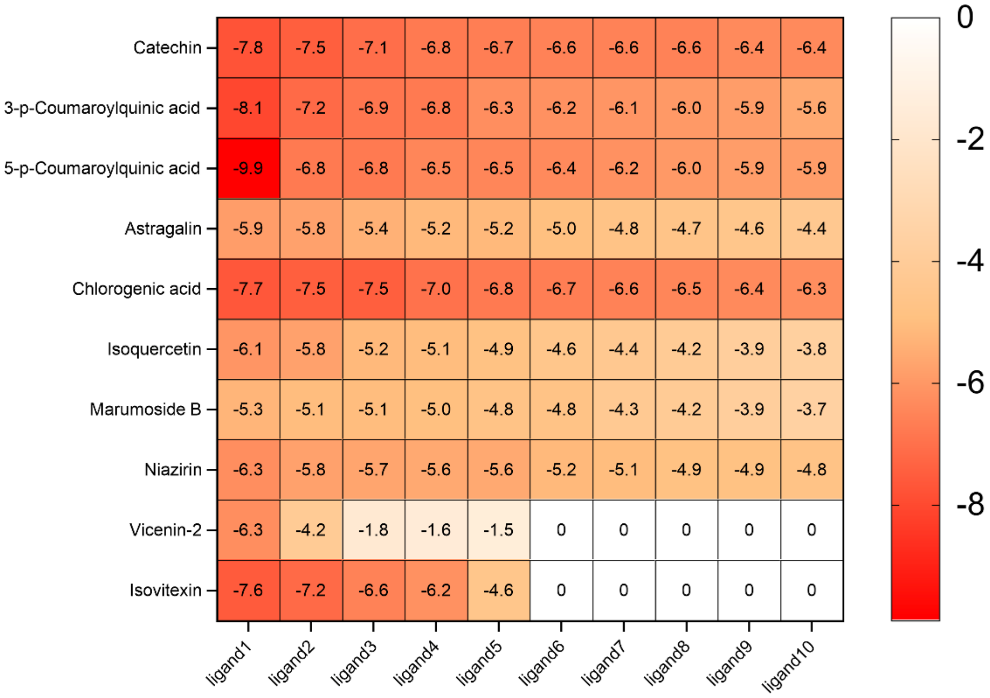

Figure.S2. Heatmap shows the different affinity between XO and molecules in MOL-TF.

Table S2. Identification of potential related metabolites and changes in the trends of metabolites after the administration of MOL-TF

No.	Ionization method	HMDB ID	Metabolite	*m/z*	Molecular formula	Trend change		
						MG vs.NG	DG vs.MG	DG vs.NG
1	ESI+	HMDB0000175	Inosinic acid	347.2363	C10H13N4O8P	↑	↓	-
2	ESI+	HMDB0000157	Hypoxanthine	135.8540	C5H4N4O	↑	↓	↓
3	ESI+	HMDB0000289	Uric acid	167.2129	C5H4N4O3	↑	↓	-
4	ESI+	HMDB0000251	Taurine	124.8808	C2H7NO3S	↓	↑	-
5	ESI-	HMDB0000192	L- cysteine	240.0127	C6H12N2O4S2	↓	↑	↓
6	ESI-	HMDB0013253	N-Acetylhistamine	152.9399	C7H11N3O	↑	↓	-
7	ESI-	HMDB0000182	L-Lysine	146.0938	C6H14N2O2	↑	↓	-
8	ESI-	HMDB0000158	Tyrosine	180.8920	C9H11NO3	↑	↓	-
9	ESI+	HMDB0004063	Metanephrine	196.7198	C10H15NO3	↑	↓	-
10	ESI+	HMDB0000819	Normetanephrine	182.5971	C9H13NO3	↑	↓	↓
11	ESI+	HMDB0000641	L-Glutamine	145.3861	C5H10N2O3	↓	↑	↓
12	ESI+	HMDB0000148	L-Glutamic acid	146.9661	C5H9NO4	↓	↑	↑
13	ESI+	HMDB0000123	Glycine	74.3807	C2H5NO2	↑	↓	-
14	ESI+	HMDB0000123	L-Arginine	173.9747	C6H14N4O2	↑	↓	↓
15	ESI-	HMDB0009427	PE(20:4(8Z,11Z,14Z,17Z)/20:0)	795.6104	C45H82NO8P	↑	↑	-
16	ESI-	HMDB0010375	CE(22:5(7Z,10Z,13Z,16Z,19Z))	698.2152	C49H78O2	↓	↑	-
17	ESI+	HMDB0007852	LysoPA(0:0/18:2(9Z,12Z))	434.1439	C21H39O7P	↑	↑	↓
18	ESI+	HMDB0003752	LysoPC(10:0/0:0)	411.4480	C18H38NO7P	↓	↑	↑
19	ESI+	HMDB0010379	LysoPC(14:0/0:0)	466.6505	C22H46NO7P	↓	↑	-
20	ESI+	HMDB0010380	LysoPC(14:1(9Z)/0:0)	464.7580	C22H44NO7P	↑	↑	-
21	ESI+	HMDB0010381	LysoPC(15:0/0:0)	481.3003	C23H48NO7P	↓	↑	↑
22	ESI-	HMDB0010383	LysoPC(16:1(9Z)/0:0)	493.1228	C24H48NO7P	↑	↑	↓

23	ESI-	HMDB0002815	LysoPC(18:1(9Z)/0:0)	520.8163	C26H52NO7P	↑	↑	-
24	ESI+	HMDB0010386	LysoPC(18:2(9Z,12Z)/0:0)	519.0122	C26H50NO7P	↓	↑	-
25	ESI+	HMDB0010388	LysoPC(18:3(9Z,12Z,15Z)/0:0)	517.4998	C26H48NO7P	↓	↑	↓
26	ESI-	HMDB0011526	LysoPE(22:6(4Z,7Z,10Z,13Z,16Z,19Z)/0:0)	525.3567	C27H44NO7P	↑	↑	-
27	ESI-	HMDB0009003	PE(18:0/20:4)	767.6885	C43H78NO8P	↑	↓	↓
28	ESI+	HMDB0001565	Phosphorylcholine	184.0728	C5H15NO4P	↑	↓	-
29	ESI+	HMDB0000259	Serotonin	175.3992	C10H12N2O	↑	↓	↓
30	ESI-	HMDB0001999	eicosapentaenoic acid	302.2734	C20H30O2	↓	↑	-
31	ESI-	HMDB0001976	docosapentaenoic acid (22n-6)	329.6426	C22H34O2	↓	↑	-
32	ESI-	HMDB0030964	linolenelaidic acid	277.4752	C18H30O2	↑	↓	-
33	ESI+	HMDB0000207	oleic acid	281.7306	C18H34O2	↓	↓	-
34	ESI+	HMDB0005060	eicosadienoic acid	308.2641	C20H36O2	↓	↑	-
35	ESI+	HMDB0002183	docosahexaenoic acid	328.3034	C22H32O2	↑	↓	↑
36	ESI+	HMDB0002823	docosatrienoic acid	333.7264	C22H38O2	↑	↓	-
37	ESI-	HMDB0002068	erucic acid	338.2088	C22H42O2	↑	↓	-
38	ESI-	HMDB0001043	arachidonic acid	304.4164	C20H32O2	↓	↓	-

PE: Phosphatidyl ethanolamines; CE: cholesterol ester; LysoPA: Lysophosphatidic Acid; LysoPC: Lysophosphatidylcholines
